# Water availability as an agent of selection in introduced populations of *Arabidopsis thaliana*: impacts on flowering time evolution

**DOI:** 10.7717/peerj.898

**Published:** 2015-04-16

**Authors:** Amanda J. Stock, Brechann V. McGoey, John R. Stinchcombe

**Affiliations:** 1 Department of Ecology and Evolutionary Biology, University of Toronto, Toronto, ON, Canada; 2 Centre for the Analysis of Genome Evolution and Function, University of Toronto, Toronto, ON, Canada

**Keywords:** Flowering time, Selective agents, Selection gradients, Water relations, Life history, Introduced species, Evolution in introduced species

## Abstract

Flowering is one of the most influential events in the life history of a plant and one of the main determinants of reproductive investment and lifetime fitness. It is also a highly complex trait controlled by dozens of genes. Understanding the selective pressures influencing time to flowering, and being able to reliably predict how it will evolve in novel environments, are unsolved challenges for plant evolutionary geneticists. Using the model plant species, *Arabidopsis thaliana*, we examined the impact of simulated high and low winter precipitation levels on the flowering time of naturalized lines from across the eastern portion of the introduced North American range, and the fitness consequences of early versus late flowering. Flowering time order was significantly correlated across two environments—in a previous common garden experiment and in environmental chambers set to mimic mid-range photoperiod and temperature conditions. Plants in low water flowered earlier, had fewer basal branches and produced fewer fruits. Selection in both treatments favored earlier flowering and more basal branches. Our analyses revealed an interaction between flowering time and water treatment for fitness, where flowering later was more deleterious for fitness in the low water treatment. Our results are consistent with the hypothesis that differences in winter precipitation levels are one of the selective agents underlying a flowering time cline in introduced *A. thaliana* populations.

## Introduction

When plants are introduced to new habitats, they may become established, and then sometimes expand their ranges beyond the area of initial introduction. Selective pressures can then act on plants in different parts of the range, leading to local adaptation. Observing this progression in introduced species offers an excellent opportunity to study evolutionary responses in colonizing populations. Many of the examples of rapid adaptation are from the invasive species literature (Sax et al., 2007), and adaptive evolution is increasingly recognized as an explanation for the ability of some introduced plants to persist and spread (Yoshida et al., 2007; Sax et al., 2007; Dlugosch & Parker, 2008). Selective agents may be the same as those found in the native range, or completely novel. Confirming major ecological agents of selection is still an active area of investigation, with more manipulative experiments necessary to confirm the forces leading to adaptation in introduced species. Here we experimentally evaluate a potential selective agent responsible for adaptive evolution of life history in introduced populations of the model plant, *A. thaliana*.

Optimizing flowering time is critical for plant species, as it will directly affect the number of seeds they can produce, and the environmental conditions their flowers and fruits will experience at critical reproductive and developmental stages (Simpson & Dean, 2002). Population differentiation in flowering time is often correlated with climatological variables, and clinal patterns in phenology are widespread (Montague, Barrett & Eckert, 2008; Keller et al., 2009). As it is such an important life history trait, adaptation in flowering time could be crucial for the success of introduced plant populations (Barrett, Colautti & Eckert, 2008; Montague, Barrett & Eckert, 2008). An unresolved challenge for plant evolutionary geneticists is understanding the selective pressures influencing time to flowering, and being able to reliably predict how it will evolve in novel environments; a task made more difficult by the high degree of plasticity involved (Stinchcombe, Dorn & Schmitt, 2004a). Flowering is also a highly complex trait controlled by multiple physiological pathways: some internally regulated and some environmentally dependent (Salome et al., 2011). Much of the past work on the transition from vegetative growth to reproduction has been accomplished using the model plant species, *Arabidopsis thaliana* (Simpson & Dean, 2002). Recent work on *Arabidopsis* has demonstrated that the same genes that underlie flowering time also influence water resource responses (Lovell et al., 2013), and that variation in water availability can impose selection on flowering time and a host of physiological traits (Kenney et al., 2014). Consequently, our knowledge of *Arabidopsis* genetics can inform ecological hypotheses and allow a more cohesive understanding of geographic variation—from the genes underlying the variation to the selection pressures behind differentiation.

In addition to its popularity as a model organism in genetics, *A. thaliana* is increasingly used to study topics in evolutionary ecology. The advantages of its wide geographic and environmental range, its experimental tractability, and the wide array of available genetic tools make it an excellent species for studies of local adaptation (Gaut, 2012). Research on *Arabidopsis thaliana* in its native European range has found clinal patterns and evidence of adaptation to climatic conditions (Stinchcombe et al., 2004b; Caicedo et al., 2004; Rutter & Fenster, 2007; Samis, Heath & Stinchcombe, 2008; Hancock et al., 2011; Fournier-Level et al., 2011). *Arabidopsis thaliana* was introduced to North America 150–200 years ago (Jørgensen & Mauricio, 2004). Despite this recent colonization, preliminary evidence for adaptive evolution has been found (Samis et al., 2012).

Samis and colleagues observed a longitudinal cline in flowering in introduced, North American *Arabidopsis*, which was parallel to that previously described in Europe and was robust to the inclusion of population structure—evidence that the cline reflects local adaptation (Samis et al., 2012). There was a correlation between a site’s longitude and the total precipitation it experienced, with eastern sites experiencing wetter conditions compared to those further west (*r* = 0.62, *p* < 0.0001) (see Mitchell & Jones, 2005 for climate data description; Samis et al., 2012). As in Europe, more coastal populations tended to flower later, while more central populations in lower precipitation conditions flowered earlier. In their analyses, Samis and colleagues observed that winter precipitation was the best explanatory factor for the North American cline. They thus hypothesized that precipitation levels through the winter are the selective agent behind population differentiation in flowering times.

Fully confirming the role of precipitation in leading to adaptive differentiation in flowering time, however, requires experimental manipulation of water availability to confirm its role as a selective agent (Wade & Kalisz, 1990). While Kenney et al. (2014) provide evidence that water availability can impose selection on flowering time using Eurasian accessions, we have no experimental data on whether it also acts on a selective agent in the introduced range. Previous studies of Arabidopsis flowering time clines and genetic associations have suggested that the genetic and geographic composition of the sample can have strong effects on the findings of a study, with variation in the strength of genetic associations or observed clines depending on what lines are included (Samis, Heath & Stinchcombe, 2008), presumably because different samples contain different genetic variation.

Because *A. thaliana* was introduced to North America only a few hundred years ago, it is possible to examine patterns of local adaptation to the new continent since then. We examined the effects of winter precipitation on flowering time and addressed two related questions: (1) Is there support for the Samis et al. hypothesis that winter precipitation could be a selective agent on flowering time variation in the introduced *Arabidopsis* range and (2) Do Kenney et al.’s findings of water availability imposing selection on life history hold for a different sample of lines and genotypes, and with water restrictions imposed during simulated winter conditions rather than warm greenhouse conditions?

## Materials and Methods

### Study species

We chose to use natural accessions of *A. thaliana* (common name: thale or mouse-ear cress) from populations in the Eastern half of the introduced North American range. We selected a subset of 199 lines used in a previous study of parallel adaptation in flowering time (Samis et al., 2012). Seeds came from accessions which had been field collected or ordered from the *Arabidopsis* stock center (*Arabidopsis* Biological Resource Center [ABRC]) and previously bulked in an environmental chamber with a vernalization period to ensure flowering.

**Figure 1:**
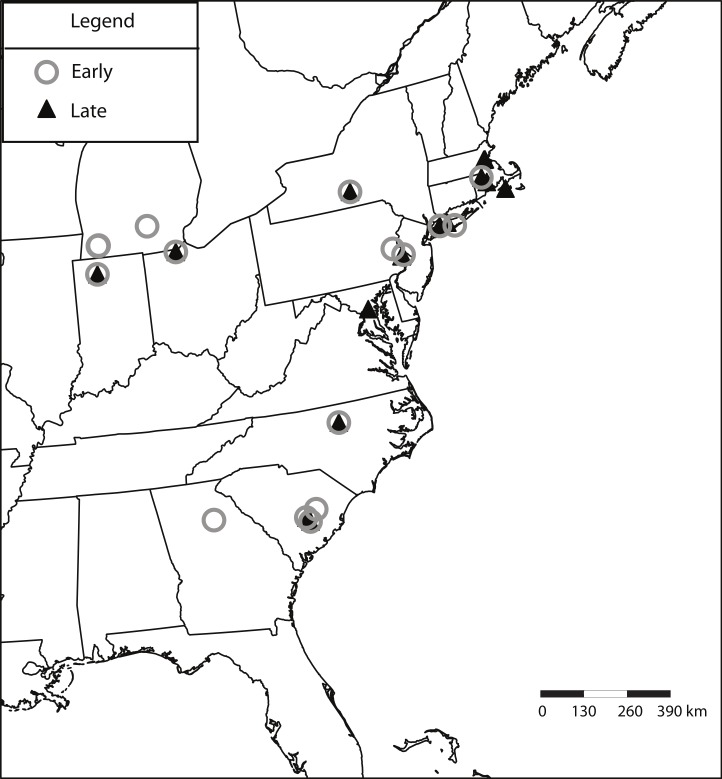
Map of origin sites of selected *A. thaliana* lines. Lines selected from the earliest flowering quintile are shown as grey circles, while lines selected from the latest flowering quintile are dark triangles.

We randomly chose 25 lines from each of the two extreme flowering time quintiles as defined by Samis et al. (2012) experiment, representing the beginning and end of the flowering time distribution (early and late flowering lines). In total, lines were drawn from 25 geographically distinct populations. The origin sites of the accessions ranged from 33.38°N to 43.27°N in latitude, and from −70.67°W to −86.62°W in longitude (Fig. 1).

### Chamber common garden

The goal of the experiment was to determine the effect of winter water levels on flowering in the introduced range. We used a 12 mL (“low”) treatment and a 24 mL (“high”) treatment administered to individual plants every other day. These parameters are similar to those used in past studies manipulating water availability (e.g., Pigliucci, Whitton & Schlichting, 1995). We applied the treatment in a split plot design because, although water was applied to individual plants, it drained down into the closed bottom of the trays where it could be absorbed by other plants in that tray. We chose to designate half the trays as “low” and half as “high” treatment level trays to prevent standing water from potentially eliminating the treatment effect.

To synchronize germination, we stratified seeds in a low concentration agar solution (0.15 mg/100 mL) for four days at 4 °C and then planted them into 4 × 8 cell trays filled with Sunshine Mix #1 soil (Sun Gro Horticulture, Agawam, Massachusetts, USA). There were ten replicate cells for each line, divided evenly between treatments: five in low water conditions and five in high. Within a treatment, lines were randomly assigned to cells in one of two blocks—which corresponded to the top shelf and bottom shelf of the chamber—to reduce microenvironmental differences. There were a total of 16 trays, eight for each treatment, and a total of 500 experimental plants plus 12 randomly chosen individuals to avoid empty cells.

The plants were germinated and grown in an environmental chamber at the Earth Sciences Centre, University of Toronto. Initially, we set the chamber to 22 °C to encourage germination, with 14 h days. Once seeds had germinated, we began the simulation of seasonally appropriate conditions (e.g., Li et al., 2006), starting with the month of October. We used the average high temperature across sites as our daytime temperature, and average low temperature as our night temperature. We set the day length as the mid-month value at the mid-longitude and latitude point for all accession sites, using standard illumination intensity (Samis et al., 2012). We adjusted the day length and temperature settings every two weeks, compressing the growing season by setting the chamber to mimic average monthly conditions from October to July.

When individual plants bolted, we recorded bolting date along with rosette diameter and the number of rosette leaves. We also noted the emergence of the first flower as flowering date. These data were collected on a daily basis, save for the earliest eight plants to bolt and flower, which were missed initially. We narrowed down the window between when they were last observed and when they were observed bolting or flowering to a period of ten days. Taking a conservative approach, we assigned all plants the last day of that window as their bolting date, or if flowering, their flowering date. When plants senesced, we collected them and recorded harvest date. Dried plants were later scored for final height, number of basal branches, and the total number of fruits—a proxy for fitness.

### Data analysis

All analyses were carried out using SAS v. 9.3 (SAS Institute Inc. 2008), with figures created in R Core Team (2014).

#### Correlations with previous flowering time data

We evaluated whether flowering times in the chamber were correlated with flowering time in the Samis et al. (2012) study using Spearman rank correlations. The significance of our results was assessed using a randomization test, described below. We also evaluated whether there was a correlation between fruit set in the chamber and precipitation at the site of origin. We included fruit set in the high and water treatments, January precipitation at the site of origin, and overall winter precipitation in these analyses.

#### Main effects of water treatment on traits and fitness

We used mixed-model ANOVA to determine the main effects of the water treatment on traits. Briefly, for each trait as a response variable, we included block and treatment as fixed effects. Block was designated as a fixed effect because our two blocks were two shelves in the chamber and did not represent random samples of spatial variation. Random effects in each model were line, line*treatment, and tray nested within treatment; the latter term was included to account for the fact that the water treatment was applied to whole trays at a time. We tested the significance of random effects using 1-tailed likelihood ratio tests (because variances cannot be less than zero), comparing -2 log likelihoods of models with and without the random effect of interest. In these models, the main effects of treatment indicate the plastic, environmental response of the trait to our experimental manipulation. The line*treatment interaction term indicates whether there is genetic variation in these plastic responses.

We estimated the denominator degrees of freedom for fixed effects using the Kenward Roger approximation (SAS, Proc Mixed). For any traits that showed differences in their means between treatments, we performed follow-up tests to determine if their relationship with fruit number differed between treatments. To do so, we modified our standard mixed model to include fixed effects of the traits and the trait by treatment interaction term.

#### Selection gradients

We estimated selection gradients for two traits, basal branch number and flowering time, in each treatment separately. We included basal branch number in our selection models because the Lande-Arnold (1983) method is sensitive to the omission of correlated traits from selection models, and several previous studies have detected selection on basal branch production (Weinig, Stinchcombe & Schmitt, 2003; Griffith, Kim & Donohue, 2004; Kelley et al., 2005; Stinchcombe et al., 2009). We estimated relative fitness by dividing individual fruit number by the mean fruit number in that treatment. We then used a mixed model with block, days to flowering, and basal branch number as fixed effects; we included line and tray as random effects. We present the fixed effect solutions for the traits as estimates of the selection gradients, as well as *P*-values from the mixed model. While the mixed model is necessary for our data to obtain proper hypothesis tests and degrees of freedom for our selection gradients, the results from a simple multiple linear regression are identical in direction and statistical significance, and are quantitatively extremely similar (parameter estimates differ by less than 1 s.e.). We also present the arithmetic mean and the standard deviation of the traits to facilitate the calculation of mean-standardized (Hereford, Hansen & Houle, 2004) and standard deviation standardized gradients (Lande & Arnold, 1983) for any subsequent meta-analyses.

#### Randomization testing

Because some of our response variables were not normally distributed, we verified the *P*-values for our hypothesis tests with randomization tests using modified versions of macros written by Cassell (2002). For the correlation between flowering time in the chamber and the rooftop common garden used by Samis et al. (2012), we examined whether the observed correlation coefficients fell into the upper or lower 2.5th percentile of the randomized distribution, which would indicate that it was more extreme than expected by chance alone. Because our goal was to compare the flowering characteristics of lines, we used inbred line means for these analyses. For the mixed models, we compared the *p*-values corresponding to the treatment, trait, or treatment*trait interaction terms to the distribution of *p*-values obtained from randomized data. These analyses were done using individual-level data. For all cases, we observed identical patterns of statistical significance using randomization, and as such we present *P*-values from standard hypothesis tests.

## Results and Discussion

### Correlations with previous flowering time data

Plants in the growth chamber flowered over 115 days, with the first plant flowering on the 37th day. Compared to previously published data on these lines, the flowering phenology observed in the growth chamber was compressed—Samis et al. (2012) report flowering time means in excess of 200 days. We had expected that plants grown in the chamber would flower earlier than the plants grown in the outdoor common garden experiment conducted by Samis et al. (2012), based on the latter having overwintered in colder conditions for much longer. Some of the plants in our experiment were able to flower during “winter” conditions because of the above 0 degree temperatures, and also experienced seasons that were artificially sped up by changing temperature and day length every two weeks.

**Figure 2:**
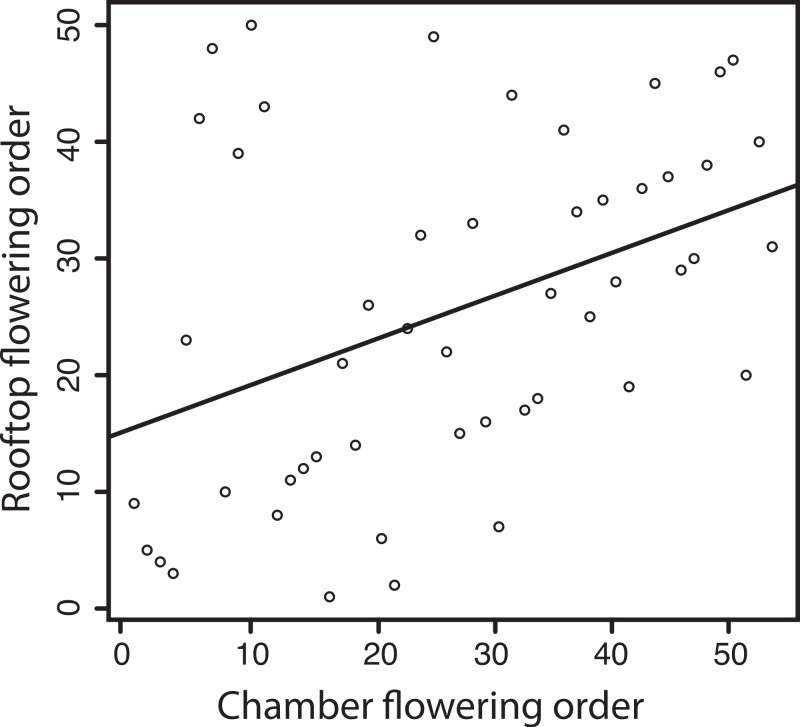
Correlation between flowering times of lines grown in our chamber experiment, and the same lines grown for a study by Samis et al. (2012) in an outdoor roof setting. For clarity, we portray the mean phenotype of lines in the chamber experiment, including both high and low water treatments.

Despite the differences in growth conditions, and mean flowering times, we observed moderate but significant rank correlations between the flowering time phenotypes observed in the growth chamber and those in the common garden (Fig. 2). Days to flower in high water and in low water were correlated with flowering time in the common garden (*ρ_s_* = 0.2966, *P* = 0.037 and *ρ_s_* = 0.3163, *P* = 0.025, for high and low respectively). Correlations across experiments were higher for bolting time (*ρ_s_* = 0.4272, *P* = 0.002 and *ρ_s_* = 0.3874, *P* = 0.0054). Comparisons between the common garden and the mean phenotypes of the chamber experiment (i.e., averaging both treatments) are of similar magnitude and significance (*ρ_s_* = 0.306, *P* = 0.03 for flowering time; *ρ_s_* = 0.417, *P* = 0.002 for bolting time). These data indicate that early and late flowering or bolting genotypes from the common garden experiment were also early and late flowering (bolting) in the growth chamber. We can conclude that there is some common underlying genetic basis for the pattern of flowering observed both outdoors and in the chamber. However, because the correlation is still significantly lower than *r* = 1, GxE interactions play a large role in determining flowering time of the lines under different conditions (Lynch & Walsh, 1998). Genotype by environment effects for flowering time in Arabidopsis are widely known (e.g., Jansen et al., 1995; Alonso-Blanco et al., 1998; El-Assal et al., 2001; Weinig et al., 2002; Ungerer et al., 2003; Juenger et al., 2005; Giakountis et al., 2010).

We failed to observe any correlation between fruit set in the chamber and precipitation levels at the site of origin (*r* < − 0.109, *P* > 0.45 for all correlations). One possible explanation for this is that our chambers did not accurately mimic natural conditions (e.g., light conditions, soil availability and pot size, our use of mid-point daylengths and temperatures, etc.). Thus while generalizing from fitness measures in the chambers to those under field conditions requires caution, comparisons between the experimental treatments *within* the chamber still allow a strong test of the hypothesis that precipitation levels could in principle impose selection on flowering time.

### Main effects of water treatment on traits and fitness

**Figure 3:**
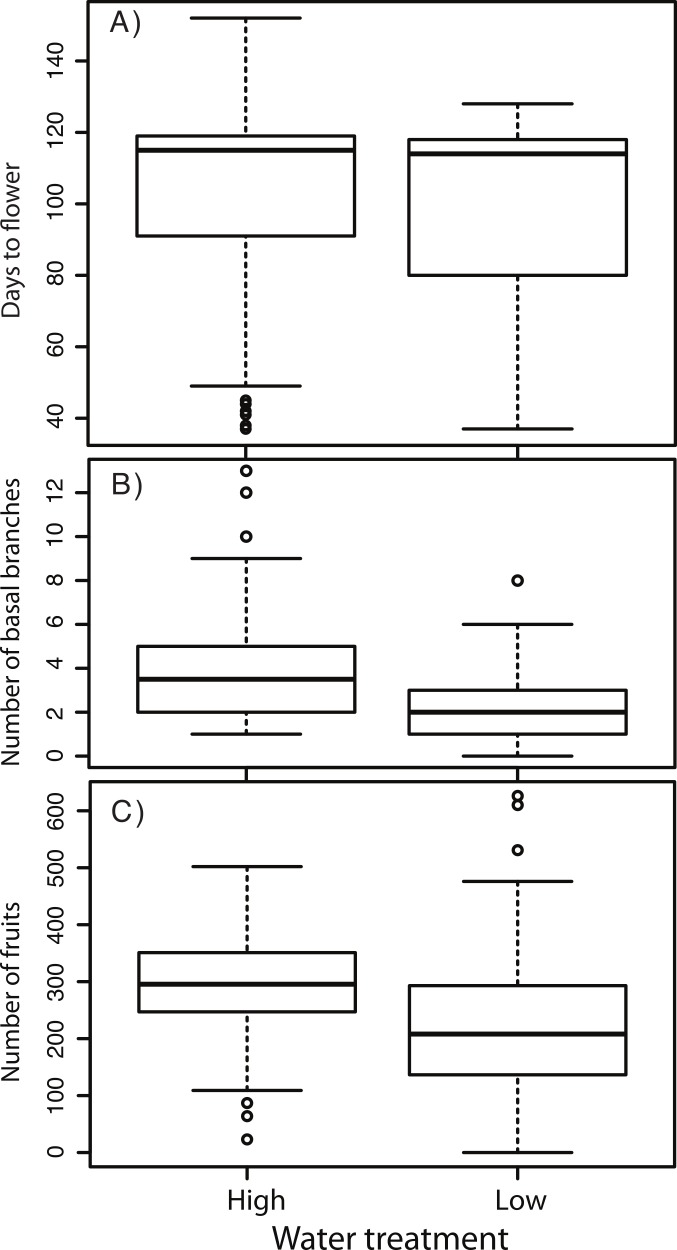
Trait and fitness differences between the high and low water treatments. (A) Flowering time (B) Number of basal branches (C) Fruit number (a proxy for fitness).

Our next goal was to evaluate the effects of the water treatment on plant size, life history, and fitness (Table 1). We found consistent effects of the water treatment on life history and morphology (least-square means ±1 standard error): plants in the low water treatment took marginally less time to flower (97.87 ± 3.91–vs–100.49 ± 3.9 days, *P* = 0.062), had significantly fewer basal (rosette) branches (2.32 ± 0.22–vs–3.69 ± 0.22), and set significantly fewer fruits (216.52 ± 11.23–vs–299.17 ± 11.39) (Fig. 3). For flowering time, our ability to resolve main effects of the treatments with the present sample sizes may be obscured by the substantial genetic variation in the plastic response to the treatment (Table 1). While on average plants accelerated their flowering under low water conditions, some lines delayed it appreciably (Fig. S1). Because of our *a priori* interest in flowering time, and the significant differences between treatments in basal branch number, we focus on these traits for subsequent analyses.

**Table 1:** Main effects of the water manipulation on life history and morphological traits. Results of mixed model ANOVAs for the six measured traits. For fixed effects, *F* statistics and *p*-values are reported. For random effects, likelihood ratio statistics and *p* values are reported.

Trait	Block	Treatment	Line	Line*Treatment
Days to flowering	*F*_1,13.4_ = 0.39, *P* = 0.54	*F*_1,15.5_ = 4.04, *P* = 0.062	*χ*^2^ = 763.9, *P* < 0.0001	*χ*^2^ = 3.6, *P* = 0.028
Basal branches	*F*_1,13.1_ = 0.08, *P* = 0.78	*F*_1,14.4_ = 26.9, *P* < 0.0001	*χ*^2^ = 67.1, *P* < 0.0001	*χ*^2^ = 1.7, *P* = 0.09
Fruit number	*F*_1,13_ = 0.65, *P* = 0.44	*F*_1,12.9_ = 36.7, *P* < 0.0001	*χ*^2^ = 43.3, *P* < 0.0001	*χ*^2^ = 0.7, *P* = 0.2
Days to bolting	*F*_1,13.3_ = 0.01, *P* = 0.93	*F*_1,11.2_ = 0.88, *P* = 0.37	*χ*^2^ = 790.3, *P* < 0.0001	*χ*^2^ = 0.2, *P* = 0.33
Rosette leaf number	*F*_1,13.4_ = 0.33, *P* = 0.57	*F*_1,17_ = 1.42, *P* = 0.25	*χ*^2^ = 333.2, *P* < 0.0001	*χ*^2^ = 5.4, *P* = 0.01
Rosette diameter	*F*_1,12.9_ = 3.67, *P* = 0.08	*F*_1,14.9_ = 1.26, *P* = 0.28	*χ*^2^ = 107.8, *P* < 0.0001	*χ*^2^ = 9.4, *P* = 0.001

### Relationships between traits and fitness

**Figure 4:**
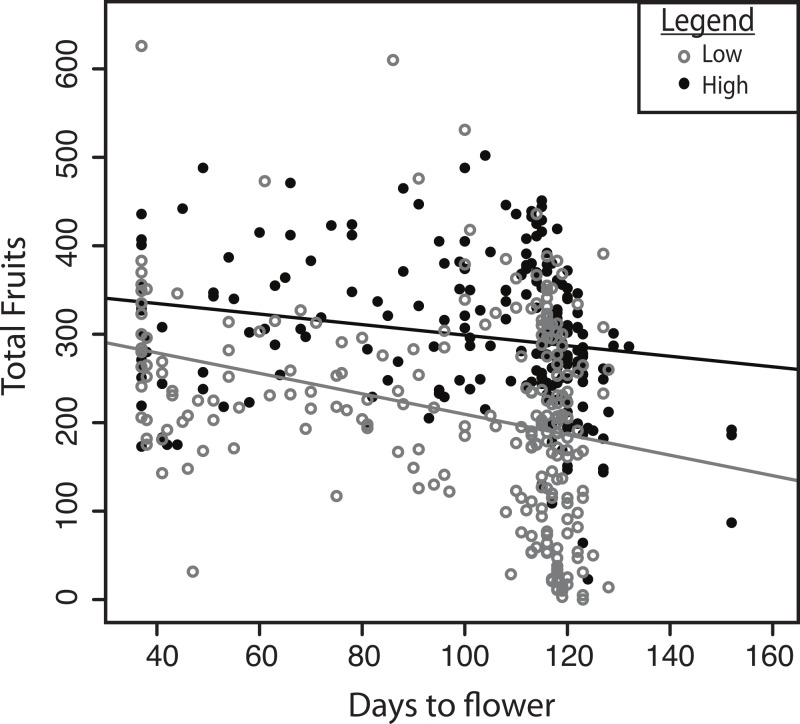
Selection on flowering time in the two experimental treatments. The total fruits produced across first flowering dates in the low (grey) and high (black) water treatments.

We next examined whether the traits that showed significant differences between treatments also had different relationships with fitness in the two treatments. The relationship between flowering time and fitness differed significantly between treatments (Fig. 4; *F*_1,420_ = 5.43, *P* = 0.02). Early flowering always led to higher fruit set, but late flowering was significantly more costly in the dry treatment. In contrast to flowering time, we observed a similar relationship between basal branch number and fruit number in both treatments (*F*_1,437_ = 0.72, *P* = 0.40). In both treatments, greater fruit number was associated with greater numbers of basal branches.

Plants can experience trade-offs in terms of investment in growth versus reproduction (Obeso, 2002). In this scenario, delayed flowering will correlate with an increased ability to accumulate resources, which can then be converted into reproductive structures and lead to high fitness. In *Arabidopsis*, however, the reproductive structures can contribute significantly more than rosettes to carbon acquisition (Earley et al., 2009), suggesting that there might not be an advantage to delaying flowering in terms of acquiring carbon. Given this, it may be advantageous for *Arabidopsis* to flower early, and have extended reproductive duration, which could explain why, in both treatments, plants that flowered earlier produced more fruits.

The more severe consequences of flowering later in the lower water treatment correspond with the phenotypic and climate characteristics of the cline that Samis et al. (2012) examined. In their study, accessions from inland sites, which are drier, tended to flower earlier, which is consistent with our findings that selection acts more strongly against late flowering under drier conditions. Harsher end of season conditions in dry sites may be the major selective pressure for earlier flowering inland. Precipitation differences could therefore lead to earlier flowering in drier inland sites in the introduced range.

### Selection gradients

Our final goal was to estimate selection gradients for flowering time and basal branch number, and thus evaluate their direct effects on fitness while accounting for their phenotypic correlation (*r* = 0.21, *P* = 0.0018 in the high water treatment; *r* = − 0.028, *P* = 0.67 in the low water treatment). Under high water conditions, we found significant selection for earlier flowering, and for greater basal branch number (Table 2). Under low water conditions, we found the same pattern: highest relative fitness was associated with early flowering and more basal branches. For both traits, selection was of much greater magnitude under low water conditions than under high water conditions: selection gradients were ∼1.7 to 1.9-fold higher under low water conditions. These results are qualitatively similar to a field study of selection *Arabidopsis thaliana*, where Kelley et al. (2005) found stronger selection on herbivore resistance traits in low-fitness environments.

**Table 2:** Selection gradients for flowering time and basal branch number in the two treatments. Unstandardized selection gradients for flowering time and basal branch number in the two watering treatments. The arithmetic mean and standard deviation of each trait in each environment are also given, to aid calculation of standardized selection gradients.

	High water				Low water			
Trait	*β*(s.e.)	*P*	$\overline{x}$	*σ*	*β*(s.e.)	*P*	$\overline{x}$	*σ*
Days to flower	−0.0030 (0.0007)	<0.0001	101.10	28.04	−0.0053 (0.0013)	<0.0001	97.80	29.09
Basal branch number	0.0414 (0.0078)	<0.0001	3.72	2.22	0.0775 (0.0221)	0.0006	2.35	1.42

In both our high and low water treatments, selection favored earlier flowering times, which raises the question of why so much variation in flowering time was seen among our lines. Selection for early flowering seems ubiquitous; a recent meta-analysis has suggested earlier flowering is generally favoured by selection, and that other constraining forces act to prevent all plants from flowering early (Munguia-Rosas et al., 2011). If flowering time really is one of the most important traits in determining fitness of a plant, why do we see so much variation among individuals from the same population experiencing the same environmental conditions? Among our populations, there were many which were polymorphic for a designation of “early” or “late” flowering, as designated by Samis et al. (2012). The cline is by no means perfect—many earlier flowering lines occur closer to the East coast, and many later flowering lines were collected from sites farther west. The possible reasons behind this within population variation include: insufficient time since colonization to respond to selection, migration from other populations, selection on correlated traits which constrains responses in flowering times, and temporally variable selection. Selection on flowering time can also be seasonally and epistatically variable in the field (Korves et al., 2007), both of which could possibly maintain variation within populations in flowering time. In addition, the timing of germination dramatically influences the fitness consequences of flowering time variation and variation at flowering time genes (Wilczek et al., 2009), suggesting that germination and seed bank dynamics could contribute to the maintenance of variation in flowering time. Selective agents outside of climatic factors may be acting on *Arabidopsis* populations, including herbivory, competition, or disturbance; different selective forces operating in opposing directions could maintain variation in flowering traits. Brachi, Morris & Borevitz (2011) describe, in detail, how broad scale climatic variation can lead to the evolution of clines in phenotypes, but micro-environmental variation in factors such as soil quality, competition, or biotic factors could lead to the maintenance of variation within populations arrayed along a cline.

Future research could focus on distinguishing among these, and potentially other, reasons why we still see such a range of flowering times maintained within populations.

## Conclusions

Understanding why plant populations evolve divergent trait values across their ranges is an important component of explaining and predicting the spread of introduced species. We have characterized one selective agent capable of acting on North American populations of *A. thaliana* in chamber conditions—winter precipitation level—as a potential cause of longitudinal variation in flowering time. Precipitation accumulated during the winter determines how moisture-limited plants will be during the spring growing season when they make the transition to reproduction and begin flowering and producing fruit. We suggest that varying patterns of precipitation across the Eastern United States could be an important selective agent acting differentially on populations across the longitudinal range of *A. thaliana*, contributing to the observed cline in flowering time.

## Supplemental Information

10.7717/peerj.898/supp-1 Table S1 Winter precipitation levels of collection sites for each selected line, along with flowering phenotypes from the low water treatment, high water treatment, and rooftop common garden of Samis et al. (2012). Rows are in order from driest to wettest average J.

10.7717/peerj.898/supp-2 Figure S1 Reaction norms of flowering time in response to the water treatment Reaction norm plot of flowering time in the two water treatments. The symbols and lines connect the mean flowering times of the same inbred lines in the two experimental treatments.

## Additional Information and Declarations

### Data Deposition

The following information was supplied regarding the deposition of related data: Data available from the Dryad Digital Repository: http://dx.doi.org/10.5061/dryad.2jk12.
