# Supplementary material for: Water availability as an agent of selection in introduced populations of Arabidopsis thaliana: impacts on flowering time evolution
Source: PeerJ. 2015 Apr 16;3:e898. doi: 10.7717/peerj.898 (PMC4406364; doi:10.7717/peerj.898)
Supplement: Table S1 [file peerj-03-898-s001.docx]

Supplemental Table 1: Winter precipitation levels of collection sites for each selected line, along with flowering phenotypes from the low water treatment, high water treatment, and rooftop common garden of Samis et al. (2012. Rows are in order from driest to wettest average January precipitation levels. Data are 30 year averages from a 0.5° grid (Mitchell & Jones, 2005).

| LineID | Latitude | Longitude | Average precipitation in January | Average total precipitation in winter months | Low treatment days to flower | High treatment days to flower | Rooftop experiment days to flower (Samis et al. 2012) |
| --- | --- | --- | --- | --- | --- | --- | --- |
| 64 | 42.500 | -84.500 | 39.0033 | 341.9866 | 40.80 | 54.20 | 203.67 |
| 52 | 41.654 | -83.536 | 43.0067 | 353.0067 | 115.80 | 120.00 | 209.00 |
| 248 | 41.654 | -83.536 | 43.0067 | 353.0067 | 119.40 | 120.80 | 209.67 |
| 249 | 41.654 | -83.536 | 43.0067 | 353.0067 | 95.60 | 95.25 | 206.20 |
| 23 | 41.295 | -86.621 | 44.9933 | 370.0334 | 102.80 | 104.80 | 204.75 |
| 51 | 41.295 | -86.621 | 44.9933 | 370.0334 | 116.80 | 118.00 | 209.33 |
| 66 | 41.295 | -86.621 | 44.9933 | 370.0334 | 90.20 | 93.00 | 203.00 |
| 77 | 41.295 | -86.621 | 44.9933 | 370.0334 | 104.40 | 100.00 | 209.00 |
| 7 | 42.440 | -76.495 | 53.0033 | 395.9966 | 119.00 | 113.80 | 205.00 |
| 49 | 42.440 | -76.495 | 53.0033 | 395.9966 | 117.80 | 118.20 | 206.00 |
| 65 | 42.440 | -76.495 | 53.0033 | 395.9966 | 119.50 | 118.80 | 209.67 |
| 72 | 42.440 | -76.495 | 53.0033 | 395.9966 | 119.80 | 122.40 | 206.00 |
| 12 | 42.116 | -86.457 | 56.0033 | 405.9834 | 116.80 | 115.60 | 206.00 |
| 15 | 42.103 | -86.485 | 56.0033 | 405.9834 | 45.00 | 53.33 | 206.33 |
| 111 | 39.030 | -76.800 | 79 | 502.9899 | 124.60 | 119.20 | 210.00 |
| 114 | 39.030 | -76.800 | 79 | 502.9899 | 123.40 | 135.40 | 209.00 |
| 45 | 40.561 | -75.396 | 83.01 | 523.0233 | 37.40 | 43.80 | 204.00 |
| 177 | 40.298 | -75.144 | 86.99 | 522.9966 | 114.00 | 115.60 | 209.20 |
| 184 | 40.298 | -75.144 | 86.99 | 522.9966 | 112.80 | 127.75 | 209.00 |
| 210 | 40.292 | -75.134 | 86.99 | 522.9966 | 117.00 | 120.50 | 209.67 |
| 236 | 40.328 | -75.054 | 86.99 | 522.9966 | 117.60 | 117.40 | 206.33 |
| 294 | 40.317 | -75.097 | 86.99 | 522.9966 | 119.80 | 120.40 | 210.50 |
| 295 | 40.292 | -75.134 | 86.99 | 522.9966 | 118.60 | 119.20 | 210.50 |
| 102 | 40.778 | -72.907 | 88.99 | 552.977 | 102.60 | 101.40 | 206.00 |
| 169 | 36.015 | -78.689 | 90.0067 | 527.0134 | 114.40 | 116.40 | 206.20 |
| 176 | 36.015 | -78.689 | 90.0067 | 527.0134 | 58.60 | 73.60 | 214.00 |
| 21 | 40.871 | -73.458 | 93.0033 | 579.0033 | 87.20 | 88.40 | 206.00 |
| 29 | 40.871 | -73.458 | 93.0033 | 579.0033 | 117.00 | 117.00 | 210.25 |
| 32 | 40.871 | -73.458 | 93.0033 | 579.0033 | 89.20 | 88.00 | 206.00 |
| 67 | 40.871 | -73.458 | 93.0033 | 579.0033 | 116.20 | 115.60 | 209.33 |
| 106 | 40.907 | -73.149 | 93.0033 | 579.0033 | 116.40 | 117.40 | 209.00 |
| 243 | 40.871 | -73.458 | 93.0033 | 579.0033 | 79.60 | 96.40 | 206.00 |
| 39 | 42.367 | -71.106 | 96.0033 | 612.0063 | 118.80 | 120.00 | 209.00 |
| 69 | 42.367 | -71.106 | 96.0033 | 612.0063 | 115.20 | 118.50 | 210.50 |
| 401 | 42.367 | -71.150 | 96.0033 | 612.0063 | 48.60 | 90.33 | 210.00 |
| 88 | 41.729 | -71.282 | 100 | 615.9907 | 76.20 | 60.50 | 210.50 |
| 89 | 41.897 | -71.431 | 100 | 615.9907 | 61.20 | 52.20 | 211.67 |
| 422 | 41.900 | -71.433 | 100 | 615.9907 | 47.00 | 61.80 | 210.50 |
| 423 | 41.900 | -71.433 | 100 | 615.9907 | 37.00 | 37.00 | 206.00 |
| 139 | 33.383 | -80.300 | 100.997 | 526.0003 | 117.80 | 113.00 | 213.00 |
| 141 | 33.383 | -80.300 | 100.997 | 526.0003 | 84.40 | 77.00 | 205.25 |
| 276 | 33.383 | -80.300 | 100.997 | 526.0003 | 112.80 | 113.00 | 203.00 |
| 122 | 33.517 | -80.417 | 102.003 | 533.0001 | 115.00 | 117.50 | 206.00 |
| 126 | 33.517 | -80.417 | 102.003 | 533.0001 | 121.40 | 120.00 | 210.50 |
| 135 | 33.517 | -80.417 | 102.003 | 533.0001 | 37.00 | 38.00 | 204.75 |
| 154 | 33.700 | -80.033 | 102.003 | 533.0001 | 115.20 | 119.00 | 206.00 |
| 159 | 33.700 | -80.033 | 102.003 | 533.0001 | 113.80 | 119.33 | 206.00 |
| 60 | 41.392 | -70.665 | 103 | 652.9963 | 117.20 | 118.20 | 209.50 |
| 121 | 33.867 | -83.551 | 126.997 | 667.9996 | 58.00 | 55.50 | 206.00 |
| 258 | 33.867 | -83.551 | 126.997 | 667.9996 | 114.20 | 114.00 | 206.33 |
