# Supplementary figures and images for: Water availability as an agent of selection in introduced populations of Arabidopsis thaliana: impacts on flowering time evolution
Source: PeerJ. 2015 Apr 16;3:e898. doi: 10.7717/peerj.898 (PMC4406364; doi:10.7717/peerj.898)

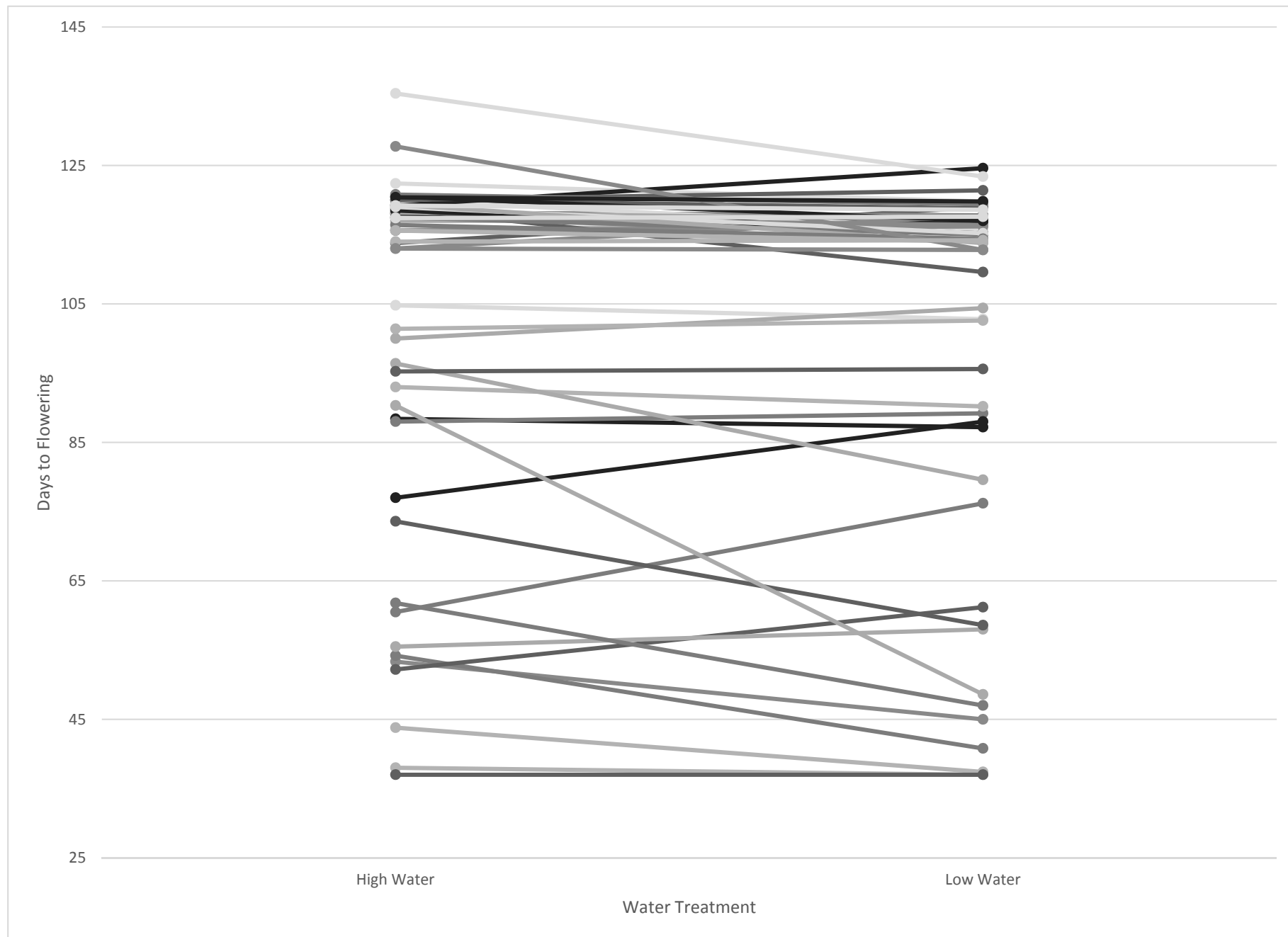

Supplement: Figure S1 — Reaction norm plot of flowering time in the two water treatments. The symbols and lines connect the mean flowering times of the same inbred lines in the two experimental treatments. [file peerj-03-898-s002.pdf]
